# Longitudinal evolution of meaning in life and its relationship with coping strategies in Spanish patients with a breast cancer diagnosis

**DOI:** 10.1007/s00520-023-07885-2

**Published:** 2023-06-26

**Authors:** Jose H. Marco, Jessica Castejón, Carmen Isern, Lola Grau, Sandra Pérez Rodríguez

**Affiliations:** 1 Department of Personality, Assessment and Psychological Treatments, Universidad de Valencia, Valencia, Spain; 2 CIBER Fisiopatología Obesidad y Nutricion (CIBEROBN), Madrid, Spain; 3 Hospital Clínica Benidorm, Alicante, Spain; 4 Escuela de doctorado, Universidad Católica de Valencia San Vicente Mártir, Valencia, Spain; 5 Universidad de Valencia, Valencia, Spain; 6 Department of Personality, Assessment and Therapeutic Interventions, Universidad Catolica de Valencia, Valencia, Spain

**Keywords:** Meaning in life, Longitudinal evolution, Breast cancer, Spanish, Coping

## Abstract

**Background:**

Changes in Meaning in life (MIL) have been shown to be particularly important when people face very stressful events such as receiving a cancer diagnosis and treatment. Active coping strategies have been related to higher levels of MIL in people with a diagnosis of cancer.

**Objective:**

To explore the evolution of MIL in a sample of cancer patients at the time of their diagnosis and three, six, and nine months after surgery, and identify the association between coping strategies three months after diagnosis (T2) and levels of MIL at the different moments in the cancer process (T1-T4).

**Methods:**

We assessed MIL at diagnosis and three, six, and nine months after surgery, and coping strategies (fighting spirit, anxious preoccupation, hopelessness, fatalism, and cognitive avoidance) three months after surgery, in 115 women with a diagnosis of Stage I-III breast cancer.

**Results:**

We found higher levels of MIL nine months after surgery, compared to previous stages. MIL correlated significantly and positively with a fighting spirit and cognitive avoidance, and negatively with hopelessness and anxious preoccupation.

**Conclusions:**

Results highlight the importance of coping in relation to meaning-making processes in cancer. Meaning-centred interventions could help patients who are in the process of coping with cancer to make sense of their lives and the experience.

## Introduction

A diagnosis of cancer is a traumatic and unexpected event [1]. The stage in which the cancer is detected and treatment begins will influence both the course of the diagnosis [2] and the presence or absence of possible recurrences [3], thus impacting physiological and psychosocial domains. In this regard, depressive, anxious, and post-traumatic symptoms or sleep problems are the most common [2]. The Spanish Society of Medical Oncology [4] reported 19.3 million new cancer cases worldwide in 2020 and 282,421 in Spain.

Meaning in life (MIL) has been shown to be particularly important when people face very stressful events [5] because an existential crisis leads to a search for meaning [6]. Consequently, MIL has been studied in various populations, such as refugees, immigrants, victims of terrorism or violence, and people diagnosed with chronic diseases, including cancer patients [7].

MIL is concerned with people’s most central, personal, and individual values. This concept includes the facets of meaning, purpose in life, and coherence of life, and it is related to the perception of responsibility in the life course [8–12]. Martela and Steger [11] suggested that MIL would be made up of three interconnected dimensions that interact to make us feel that life has meaning. On the one hand, the Coherence dimension is the cognitive component of MIL, and it is defined as the degree to which people feel that the world in which they live is organized, structured, predictable and explainable as a whole. The second dimension is Purpose, which is the motivational dimension, and it refers to the way people experience their life as oriented and guided by objectives and vital goals. The third dimension is Significance, the affective dimension, which refers to the feeling that one's life has inherent value and is worth living. From this perspective, having a high level of MIL would contribute to proposing and achieving vital objectives that guide and give importance to one's life within a coherent and organized whole. Thus, people who experience MIL are better prepared to successfully tackle life’s circumstances, and they have a strong sense of autonomy, self-determination, and purpose in life [13].

An important distinction has to do with the difference between the concepts of MIL and meaning of life. The latter refers to the metaphysical question of why the human race exists in general [8]. In addition, Frankl called the meaning of life a supra-meaning or ultimate meaning related to existential grounds and outside our whole being, i.e., through faith [9]. In this study, we will explore MIL.

A cancer diagnosis often affects MIL by challenging the patient’s set of personal beliefs [6] and questioning the life purpose, identity, and actions triggered by personal values ​​[13] as patients try to incorporate the experience of the disease into their life scheme [14, 15]. MIL may change through a process of meaning-making, defined as cognitive efforts to reduce the discrepancy between one's appraisal of a stressor and one's global meaning (i.e., beliefs, goals, and MIL) [15]. Park’s integrated meaning-making model [15] proposes that stressful life events (e.g., a cancer diagnosis) lead to a discrepancy between the global meaning -an individual’s general orienting system, including goals, feelings, global beliefs, and general concepts through which individuals interpret their world’s experiences- and the situational meaning -meaning in the context of a particular environmental encounter (the cancer diagnosis) that challenges a person's MIL and leads to meaning-making efforts. Successful meaning-making efforts result in a greater or restored sense of MIL and reduced distress. Failure to achieve meaning from the experience will lead to distress and psychopathology [15]. Studies have shown that, in advanced cancer patients, finding meaning leads to an increase in the assessment of their lives as positive and worthy [16].

Studies in cancer patients have found that low MIL is related to distress and a low sense of coherence, according to the results of a meta-analysis [17]. Borreani et al. [7] found a negative relationship between MIL and fear of recurrence, depression, and anxiety in a sample of 75 haematological cancer patients, and a recent systematic review found that MIL was correlated with acceptance of cancer but did not predict the acceptance of the disease [18]. In a multinational study of patients with cancer [19], the results did not show differences in levels of MIL across countries. Moreover, low MIL was predicted by physical symptoms, psychological distress, and existential and financial concerns, whereas higher MIL was associated with being married, being optimistic, and having higher education.

As mentioned above, the meaning-making process may change throughout the cancer process [15]. However, few studies have explored the evolution of MIL longitudinally during the cancer experience. Of them, Campo et al. [20], in a sample of 254 post-stem cell cancer survivors, found that resilience predicted increases in MIL. Another study, carried out with 108 Chinese terminally ill patients admitted to a palliative care unit [21], showed decreases in MIL from the time of admission to the unit. Moreover, meaning decreased with the increase in depression and pain levels. Moreover, Lin et al. [22] conducted a longitudinal study with 160 Taiwanese cancer patients undergoing radiotherapy whose meaning in life was assessed one week before and one week after radiotherapy. The authors observed higher levels of meaning in life in patients after treatment than before it. Moreover, they found lower levels of meaning in life in Stage IV patients who were unable to receive surgery and in patients who experienced greater distress after radiotherapy. However, of the studies mentioned, only the work by Lin et al. [22] explored the trajectories of MIL throughout the different phases of the cancer experience, in this case, only before and after radiotherapy treatment.

The presence of MIL has been related to coping strategies during the diagnosis and treatment stages of cancer in a scarce number of studies. Jim et al. [23] found that positive coping strategies (e.g. acceptance, positive reinterpretation, active coping, seeking social support, and less denial) predicted higher levels of MIL in breast cancer patients two years after diagnosis. Krok and Telka [24] found that meaning had indirect effects on psychological well-being through the use of problem-, emotion-, and meaning-focused coping strategies in gastric cancer patients, and Krok et al. [25] found that MIL moderated the relationship between illness perception and affective symptoms through problem- and meaning-focused coping. In a meta-analysis, Quinto et al. [18] found that acceptance of cancer was associated with higher MIL.

However, to our knowledge, there are no studies that have explored MIL trajectories throughout the cancer process, that is, after diagnosis, surgery, and oncology treatments, and none have been carried out in Spanish non-metastatic breast cancer patients. Moreover, few studies have explored the relationship between coping at cancer diagnosis and the later MIL status. Thus, the main objective of this study was to explore the evolution of MIL in a sample of cancer patients at the time of their diagnosis (T1) and at different time points after surgery (3, 6, and 9 months after surgery, T2-T4 respectively). A second objective was to identify the association between coping strategies three months after diagnosis (T2) (fighting spirit, anxious preoccupation, hopelessness, fatalism, and cognitive avoidance) and levels of MIL at the different moments in the cancer process (T1-T4).

## Method

### Participants

We approached a consecutive sample of 139 patients with a diagnosis of Stage I-III non-metastatic breast cancer. Stage IV patients were not included in the study because they followed different treatment protocols and had a lower life expectancy. We recognize the importance of death expectancy in the consideration of meaning in life. However, we were specifically interested in non-metastatic breast cancer patients. Thus, the final sample was composed of 115 women with breast cancer who agreed to participate, signed the written inform consent, and actively answered the subset of questionnaires that made up the first assessment in this study. This research was carried out between June 2015 and December 2016, and it had a duration of 1.5 years, during which the participants attended the Functional Unit of Breast Pathology of the Hospital Universitario y Politécnico La Fe de Valencia, Spain. All the participants were between 37 and 92 years old (*M* = 59.99, *SD* = 10.74). Table 1 shows other sociodemographic characteristics.

**Table 1:** Sociodemographic characteristics of the sample

	Num. (%)
Age ranges	
25–40	5 (4.3%)
41–50	18 (15.7%)
51–65	57 (49.6%)
66–75	23 (20%)
> 75	12 (10.4%)
Marital status	
Single	15 (13%)
In couple	75 (57%)
Divorced	9 (7.8%)
Widow	16 (13.9%)
Level of Studies	
No formal education	4 (3.5%)
Primary	40 (34.8)
High school	37 (32.2%)
Higher education	34 (29.6%)
Occupation	
Domestic labour	68 (59.2%)
Work outside the home	47 (20.8%)
Retired	23 (20%)
Mental health prior diagnosis	
No diagnosis of mental disorder	100 (87%)
Major depressive disorder	8 (7%)
Anxiety disorder	1 (0.9%)
Prolonged grief disorder	3 (2.6%)
Bipolar disorder	1 (0.9%)
Difficulties in primary support network	2 (1.7%)

Regarding the mental health of the patients, *n* = 100 (87%) did not have a diagnosis of psychological disorders prior to the cancer diagnosis, compared to 15 who did (see Table 1).

Regarding the medical condition of the participants, *n* = 6 (5.2%) had previously been diagnosed with breast cancer. In terms of the cancer treatment, *n* = 115 (100%) underwent surgical treatment, which consisted of radical surgery for *n* = 33 (28.7%) patients and conservative surgery for the other 82 (71.3%). The majority of the patients received an adjuvant treatment or a combination of them after surgery during the time of the study: neoadjuvant only *n* = 2 (1.9%); adjuvant only *n* = 97 (84.3%); and both *n* = 16 (13.9%). As for the type of treatment, *n* = 72 (62.6%) received chemotherapy, *n* = 41 (35.7%) radiotherapy, and *n* = 2 (1.7%) hormonal treatment. No information was collected on whether participants had or had not previously had other types of cancer.

### Instruments

In relation to Meaning and Purpose in Life, the Purpose In Life Inventory—10 items (PIL-10) [26, 27], whose objective is to measure meaning and purpose in life, was used. It is a reduced version that contains ten items rated on a Likert-type scale, with seven response levels. The distribution of the dimensions assessed is: (1) enthusiasm/boredom, (2) enthusiasm for life, (3) presence of life goals, (4) daily novelty, (5) desire for more lives, (6) post-retirement activity, (7) good aspects of life, (8) having a reason to be alive, (9) ability to find meaning, and (10) existence of goals/life purpose. The total score ranges from 10 to 70, with higher scores corresponding to greater meaning/purpose in life. The validation in Spanish preserves good internal consistency (α = 0.88) and a high reliability coefficient in the sample (α = 0.85) (García-Alandete et al., 2013). In our sample, we obtained adequate internal consistency (α = 0.86).

Coping was assessed using the MINI-Mental Adjustment to Cancer Scale (Mini-MAC) [28], which is one of the most widely used instruments to assess coping in cancer and evaluate behavioural and cognitive responses to cancer. It consists of 29 items rated on a 4-point scale, and it includes five subscales: fighting spirit (the tendency to confront and actively face illness), anxious preoccupation (the tendency to experience illness as a source of marked anxiety and tension), fatalism (the tendency to have a resigned and fatalistic attitude towards illness), hopelessness–helplessness (the tendency to adopt a pessimistic attitude toward illness), and cognitive avoidance (the tendency to avoid direct confrontation with illness-related issues). A confirmatory factor analysis of the reliability levels of the Spanish adaptation [29] confirmed a five-factor solution for the instrument. This instrument was used at T2, three months after the surgery, following the recommendations of Greer [30], thus allowing the assessment of the more enduring psychological coping responses, which, according to the author [30], stabilize three months after diagnosis.

### Procedure

**Fig. 1:**
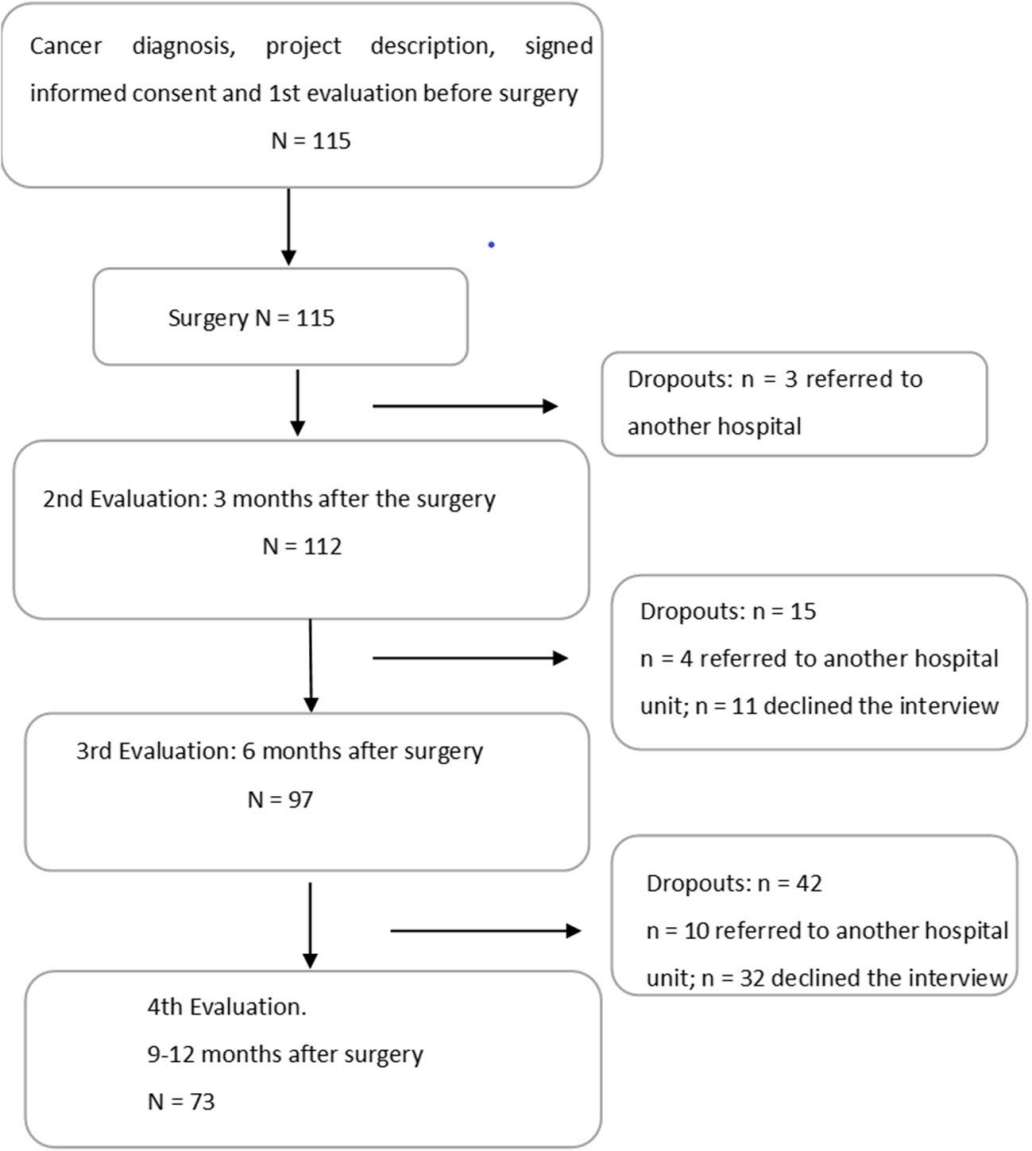
Flowchart

In this longitudinal study, data were collected at four time points. The interviews took place after the patients’ consultation with their surgeon, which was always conducted by the same professional and using the same structure in order to reduce possible bias. The first assessment (T1) was administered when the sample was informed of the diagnosis of cancer, after the second visit to the surgeon, with *n* = 115 (100%) participants being assessed (Fig. 1). The second assessment time (T2) took place three months after the surgery or 4–5 months after the diagnosis, with *n* = 112 (97.4%) patients assessed at this time point. The third time point (T3) was six months after the surgery, and it included *n* = 97 (84.3%) patients. The fourth time point (T4) took place nine months after the surgery, and *n* = 73 (63.5%) participants were assessed.

The current investigation was evaluated and approved by the Clinical Research Ethics Committee of the Health Research Institute of the Hospital Universitario y Politécnico La Fe de Valencia. Therefore, it respects the ethical and moral principles for clinical research in humans, included under code 2015/0317.

### Statistical analysis

Regarding the evaluation of the demographic and descriptive variables, their respective means, descriptive statistics, and frequencies were calculated. To compare differences in MIL between the age and disease stage groups, we carried out ANOVAS and Bonferroni post-hoc tests. For the objectives and proposed hypotheses, the Multivariate Analysis and the Repeated Measures Analysis were used. To analyse the relationship between coping strategies and MIL, Pearson bivariate correlations were conducted. SPSS package version 26 was used.

## Results

First, we did not find statistically significant differences in MIL among the age groups in T1 to T4 (T1: F = 2.32, *p* = 0.062; T2 = 1,71, *p* = 154; T3: F = 0.89, *p* = 471; T4: F = 2.69, *p* = 0.04). Although the total test showed statistically significant differences at T4, Bonferroni post-hoc analysis did not reveal statistically significant differences between specific age groups (*p* = 0.07 to 1.00).

The results obtained showed statistically significant differences between levels of MIL across the different time points (F(3,70) = 6.90, *p* = 0.001). Means, standard deviations, and Bonferroni post hoc analyses are shown in Table 2. Post hoc analyses revealed statistically significant differences between levels of MIL in T4, nine months after surgery (*M* = 63.23, *SD* = 0.57), and levels in T2 and T3 (surgery, *M* = 60.62, *SD* = 0.68, *p* = 0.000 and six months after surgery *M* = 60.99, *SD* = 0.80, *p* = 0.000), with higher levels in T4. However, although a slight increase in MIL is observed between T1 and T4, there were no statistically significant differences in the MIL scores between these two phases (T1: *M* = 61.82, *SD* = 0.97, *p* = 0.35) and T4 (Table 2; Fig. 1).

**Table 2:** Differences in MIL across stages of the study

*N* = 115	T1	T2	T3	T4				
	*M (SD)*	*M (SD)*	*M (SD)*	*M (SD)*	*F*	*df*	η_p_^2^	Bonferroni post-hoc
MIL	61.82 (8.33)	60.62 (5.67)	60.99 (6.83)	63.23 (4.95)	6.90***	3, 70	.18	2 < 4; 3 < 4

T1: after diagnosis: T2: 3 months after surgery; T 3: 6 months after surgery; T4: 9–12 months after surgery. *MIL* meaning in life

***p < 0.001

**Fig. 2:**
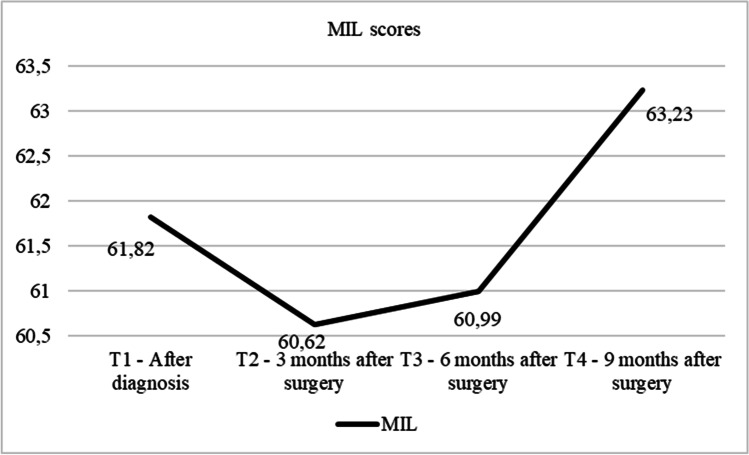
Graph of the distribution of MIL in each phase of the study

Figure 2 shows a non-significant decrease in meaning in life after the cancer diagnosis (T1) that lasted up to three months after completing the medical treatment (T2). From this point on, meaning in life increased progressively and significantly until nine months after surgery (T4).

As for correlations between coping strategies and MIL at the four time points when they were assessed (T1-T4), we found negative and statistically significant correlations between hopelessness and MIL at T2 (*r* = -0.28, *p* = 0.0017, at T3 (*r* = -0.37, *p* = 0.002) and at T4 (*r* = -0.36, *p* = 0.002). Anxious preoccupation was negatively and significantly correlated with MIL at T3 and T4 (*r* = -0.31, *p* = 0.007 and *r* = -0.26, *p* = 0.028), respectively). Fighting spirit was significantly and positively associated with MIL in all the phases: T1 (*r* = 0.32, *p* = 0.006); T2 (*r* = 0.29, *p* = 0.012); T3 (*r* = 0.27, *p* = 0.021); and T4 (*r* = 0.27, *p* = 0.023), and cognitive avoidance was positively and significantly correlated with MIL at T2 (*r* = 0.30, *p* = 0.010), T3 (*r* = 0.34, *p* = 0.003) and T4 (*r* = 0.36, *p* = 0.002). We did not find any statistically significant correlations between fatalism and MIL (Table 3).

**Table 3:** Correlations between coping strategies at T2 (3 months after diagnosis) and MIL

	MIL T1	MIL T2	MIL T3	MIL T4
Hopelessness	-.16	-.28*	-.37**	-.36**
Anxious preoccupation	-.08	-.17	-.31**	-.26*
Fighting spirit	.32**	.29**	.27*	.27*
Cognitive avoidance	.21	.30**	.34**	.36**
Fatalism	.10	.14	.03	.003

*p < 0.05; **p < 0.01; *** p < 0.001

## Discussion

The first aim of this study was to explore the evolution of MIL in a sample of 115 patients with a diagnosis of breast cancer from the time of their diagnosis and three, six, and nine months after surgery. A second objective was to identify the association between coping strategies three months after surgery (T2) (fighting spirit, anxious preoccupation, hopelessness, cognitive avoidance, and fatalism) and levels of MIL at the different moments in the cancer process (T1-T4).

The results of the study showed that, in our sample, the evolution of meaning in life changes across the different time points in the cancer diagnosis and treatment. Specifically, MIL increased significantly nine months after surgery, compared to the assessments at three and six months. However, MIL at three, six, and nine months remained similar to the levels observed before the surgery, right after the cancer diagnosis.

A diagnosis of cancer is a traumatic and unexpected event that threatens patients’ survival and challenges their personal beliefs, life purposes, and personal values ​​[6]. Thus, it seems logical that the process of finding meaning would change throughout the experience of cancer, especially in the first year after the diagnosis when the first phases occur -diagnosis, surgery, chemotherapy, and radiotherapy ​​[21]. In our sample, which consists of patients who have Stage I to III cancer or curable cancer, MIL increased after the active treatment had ended (T4), in comparison with previous phases. Previous studies focused on posttraumatic reactions to a cancer diagnosis have found higher levels of distress after diagnosis than after surgery or active treatments, thus indicating that the first moments in the cancer process seem to be the most distressing, and when the treatment is over, the patients seem to recover psychologically [29]. Similar to our results related to MIL, Lin et al. [22] observed higher levels of MIL in patients after treatment with radiotherapy than before it in earlier phases, but these levels were lower in patients with cancer in a non-curable stage. In the same direction, Huamani and Arohuanca [31] found that 74% of their sample had reached medium or high levels of meaning after treatment. These results could indicate that, during the more active phases of cancer (diagnosis and active treatment), patients make an effort to manage and cope with the high demands of the disease (accept the reality of the diagnosis, cope with the surgery, pain, and secondary effects of treatment). Once the active treatment has ended, the patients can focus on the evaluation of their process and, thus, sometimes find meaning in the experience. Although studies on the evolution of MIL are scarce, previous studies have documented the relationship between higher posttraumatic growth up to 24 months after diagnosis and higher levels of meaning and active coping strategies [32].

As for the second objective, we explored the association between the more enduring coping strategies assessed three months after diagnosis (fighting spirit, anxious preoccupation, hopelessness, cognitive avoidance, and fatalism) and levels of MIL at the different time points in the cancer process (T1-T4). Hopelessness three months after diagnosis was negatively and significantly related to lower levels of MIL three, six, and nine months after surgery. That is, when patients used a coping strategy characterized by negative thoughts related to despair and lack of hope three months after the diagnosis, they were more likely to show low levels of MIL throughout the different stages of the cancer. In addition, anxious preoccupation was also negatively associated with MIL. When patients faced the illness with cognitions of preoccupation, MIL was also lower at six and nine months, thus indicating that strategies focused on anxiety and rumination are related to problems with finding meaning in the experience. In contrast, a fighting spirit was positively associated with higher MIL. Patients who showed a fighting spirit or viewed the illness as a challenge, looking for information about what they can do and adopting realistic but active strategies to manage, reduce, or solve the problem -in this case the cancer, were more likely to find meaning in the experience in all the phases. Previous work has documented the relationship between MIL and coping and their mediational role in distress reactions to cancer [33]. Specifically, some studies have found support for the role of active coping as a protective factor against distress [29, 34]. Although there is consensus about the adaptive role of a fighting spirit or the maladaptive role of anxious preoccupation and hopelessness in coping with cancer [29], there is contradictory evidence about the adaptive/maladaptive role of cognitive avoidance and fatalism [35].

In our study, surprisingly, cognitive avoidance, or not thinking about the cancer and avoiding places or conversations that act as reminders of the illness, was related to higher levels of MIL from six to nine months after diagnosis. Although previous research and meta-analyses have found cognitive avoidance to be more related to lower psychological well-being, it seems that the adaptiveness depends on the stage of the cancer, given that it is more maladaptive in women not undergoing treatment or in more advanced stages of the disease [36]. In our sample of patients with breast cancer in curable stages, the use of cognitive avoidance, or not thinking about the illness, may have given patients the opportunity to move on with the illness and activate a fighting spirit. In fact, these two strategies were positively and significantly related in our study (*r* = 0.37, *p* = 0.001). We did not find a relationship between the fatalism coping strategy and MIL, although previous work has related this strategy to a maladaptive coping profile in Western countries [29].

As we highlighted in the introduction section, few studies have explored the predictive role of coping in meaning in life in cancer patients. Jim et al. [23] found that positive coping strategies predicted higher levels of MIL in breast cancer patients two years after diagnosis, and Krok et al. [25] found associations between MIL and problem-focused coping strategies, thus supporting our results linking adaptive active coping strategies to meaning-making from the cancer experience.

### Limitations and future directions

Our work has some limitations. First, our sample was composed of Spanish women who were breast cancer patients in stages I-III, and so the results cannot be generalized to patients from other countries, to men, or to patients with other types of cancer or stages of the cancer. Second, our study was longitudinal, and participants were assessed at different moments in the cancer illness (3, 6, and 9 months after surgery). However, these assessment time points are temporal moments that could represent different moments in the illness process for the patients (for example, some patients could be receiving chemotherapy at T3, whereas others could be receiving radiotherapy or hormonal therapy). These differences could affect the levels of MIL because the impact of the secondary effects of treatment could influence the meaning-making process. Third, we assessed patients until nine months after surgery, but we did not assess meaning in life later in the disease process. Finally, the small sample size and the limited time frame kept us from implementing other statistics that might have shed more light on the results. Future research should explore, from a longitudinal perspective, meaning in life in samples with different characteristics, such as other types of cancer or in metastatic stages of the disease.

Despite these limitations, our study is the only one that addresses meaning in life longitudinally through the different stages of the cancer experience in Spanish breast cancer patients, and it sheds light on the evolution of the construction of meaning in this population.

## Conclusions

The evolution of MIL changes across the different time points in the cancer diagnosis and treatment.

MIL reduces during the cancer treatment and increases nine months after surgery.

Hopelessness, and anxious preoccupation are related negatively to MIL across stages, and fighting spirit and cognitive avoidance are relates positively.

### Clinical implications

Results highlight the importance of focusing on coping in relation to meaning-making processes in cancer. In this regard, meaning-centred interventions could help patients who are in the process of coping with the cancer experience to acquire more active coping strategies by identifying sources of meaning and vital goals and, when possible, finding meaning in the experience.

## Data Availability

The datasets generated by the survey research during and/or analyzed during the current study are available in the Harvard Dataverse repository: 10.7910/DVN/MSNZSP, Harvard Dataverse.

## References

[1] Andrykowski MA, Steffens RF, Bush HM, Tucker TC (2015). Lung cancer diagnosis and treatment as a traumatic stressor in DSM-IV and DSM-5: prevalence and relationship to mental health outcomes. J Trauma Stress.

[2] Gori A, Topino E, Sette A, Cramer H (2021). Pathways to post-traumatic growth in cancer patients: moderated mediation and single mediation analyzes with resilience, personality, and coping strategies. J Affec Disord.

[3] Ogińska-Bulik N (2018). The role of ruminations in relation between personality and positive posttraumatic changes resulting from struggling with cancer. Health Psychol Rep.

[4] Spanish Society of Medical Oncology (2021) Cancer figures in Spain . Cited 2022 Sept 1. https://seom.org/images/Cifras_del_cancer_en_Espnaha_2021.pdf

[5] King LA, Hicks JA (2021). The science of meaning in life. Annu Rev Psychol.

[6] Elekes S (2017). The relation of perceived meaning of life, meaning of illness and anxious-depressive symptoms among cancer patients. Eur J Ment Health.

[7] Borreani C, Alfieri S, Farina L, Bianchi E, Corradini P (2020). Fear of cancer recurrence in haematological cancer patients: exploring socio-demographic, psychological, existential and disease-related factors. Supp Care Cancer.

[8] Ebersole P, DeVore G (1995). Self-actualization, diversity, and meaning in life. J Soc Behav Personal.

[9] Frankl VE (1969). The will to meaning: principles and application of logotherapy.

[10] Park CL, Hanna D Meaning, spirituality, and perceived growth across the cancer continuum: a positive psychology perspective. In: Psychological aspects of cancer. Cham: Springer; 2022. p. 91–108

[11] Martela F, Steger MF (2016). The three meanings of meaning in life: Distinguishing coherence, purpose, and significance. J Posit Psychol.

[12] Carreno DF, Eisenbeck N, Hicks JA, Holte P, Kim J, Li Z, Schlegel RJ, Shanahan C, Martela F, Zhang H (2022). Experiential appreciation as a pathway to meaning in life. Nat Hum Behav.

[13] Frankl VE (2006). The unheard cry for meaning.

[14] Britt KC, Acton G (2022). Exploring the meaning of spirituality and spiritual care with help from Viktor Frankl. J Holist Nurs.

[15] Park CL (2010). Making sense of the meaning literature: an integrative review of meaning making and its effects on adjustment to stressful life events. Psychol Bull.

[16] Bernard M, Berchtold A, Strasser F, Gamondi C, Borasio GD Meaning in life and quality of life: palliative care patients versus the general population. BMJ Support Palliat Care. 2020;bmjspcare-2020–002211. 10.1136/bmjspcare-2020-00221 10.1136/bmjspcare-2020-002211 PMC11671900 32631960

[17] Winger JG, Adams RN, Mosher CE (2016). Relations of meaning in life and sense of coherence to distress in cancer patients: a meta-analysis. Psychooncology.

[18] Quinto RM, De Vincenzo F, Campitiello L, Innamorati M, Secinti E, Iani L (2022). Meaning in life and the acceptance of cancer: a systematic review. Int J Environ Res Public Health.

[19] Gravier AL, Shamieh O, Paiva CE, Perez-Cruz PE, Muckaden MA, Park M, Bruera E, Hui D (2020). Meaning in life in patients with advanced cancer: a multinational study. Support Care Cancer.

[20] Campo RA, Wu LM, Austin J, Valdimarsdottir H, Rini C (2017). Personal resilience resources predict post-stem cell transplant cancer survivors' psychological outcomes through reductions in depressive symptoms and meaning-making. J Psychosoc Oncol.

[21] Yang Y, Zhao X, Cui M, Wang S, Wang Y (2021). Longitudinal changes in spiritual well-being and associations with emotional distress, pain, and optimism-pessimism: a prospective observational study of terminal cancer patients admitted to a palliative care unit. Support Care Cancer.

[22] Lin HS, Wang C, Chou FH, Tang PL (2021). Tumor origin and symptom distress of radiotherapy affected fluctuation of purpose in life for cancer patients. Psychol Health Med.

[23] Jim HS, Richardson SA, Golden-Kreutz DM, Andersen BL (2006). Strategies used in coping with a cancer diagnosis predict meaning in life for survivors. Health Psychol.

[24] Krok D, Telka E (2019). The role of meaning in gastric cancer patients: relationships among meaning structures, coping, and psychological well-being. Anxiety Stress Coping.

[25] Krok D, Telka E, Zarzycka B (2019). Illness perception and affective symptoms in gastrointestinal cancer patients: a moderated mediation analysis of meaning in life and coping. Psychooncology.

[26] Crumbaugh JC, Maholick LT (1969). Manual of instructions for the purpose in life test.

[27] García-Alandete J, Rosa E, Sellés P (2013). Estructura factorial y consistencia interna de una versión española del Purpose-In-Life Test [Factorial structure and internal consistency of a Spanish version of the Purpose-In-Life Test]. Univ Psychol.

[28] Watson M, Law M, Dos Santos M, Greer S, Barich J, Bliss J (1994). The Mini-MAC: Further development of the manual of the mental adjustment to cancer scale. J Psychos Oncol.

[29] Andreu Y, Galdón MJ, Durá E, Martínez P, Pérez S, Murgui S (2012). A longitudinal study of psychosocial distress in breast cancer: prevalence and risk factors. Psychol Health.

[30] Greer S (1991). Psychological response to cancer and survival. Psychol Med.

[31] Huamani JC, Arohuanca M (2019). Meaning of life in patients diagnosed with cancer. Person.

[32] Bellizzi KM, Blank TO (2006). Predicting posttraumatic growth in breast cancer survivors. Health Psychol.

[33] Hullmann SE, Robb SL, Rand KL (2016). Life goals in patients with cancer: a systematic review of the literature. Psychooncology.

[34] Chirico A, Serpentini S, Merluzzi T, Mallia L, Del Bianco P, Martino R, Trentin L, Bucci E, de Laurentiis M, Capovilla E, Lucidi F, Botti G, Giordano A (2017). Self-efficacy for coping moderates the effects of distress on quality of life in palliative cancer care. Anticancer Res.

[35] Cheng CT, Ho SM, Lai Y, Zhang Q, Wang GL (2021). Coping profiles predict long-term anxiety trajectory in breast cancer survivors. Support Care Cancer.

[36] Kvillemo P, Bränström R (2014). Coping with breast cancer: a meta-analysis. PLoS One.

